# The impact of different corneal refractive surgeries on binocular dynamic visual acuity

**DOI:** 10.3389/fnins.2023.1142339

**Published:** 2023-03-03

**Authors:** Yuexin Wang, Yining Guo, Yuanting Li, Yu Zhang, Yifei Yuan, Tingyi Wu, Yueguo Chen, Xuemin Li

**Affiliations:** ^1^Department of Ophthalmology, Peking University Third Hospital, Beijing, China; ^2^Beijing Key Laboratory of Restoration of Damaged Ocular Nerves, Beijing, China

**Keywords:** myopia, dynamic visual acuity, laser-assisted sub-epithelial keratomileusis, femtosecond laser-assisted *in situ* keratomileusis, small incision lenticule extraction

## Abstract

**Purpose:**

To investigate the influence of different corneal refractive surgeries on dynamic visual acuity (DVA), and explore its potential influence factors.

**Methods:**

This was a prospective non-randomized study. Adult myopic patients undergoing bilateral laser-assisted sub-epithelial keratomileusis (LASEK), femtosecond laser-assisted *in situ* keratomileusis (FS-LASIK), or small incision lenticule extraction (SMILE) with Plano refraction target were enrolled. Uncorrected and corrected distance visual acuity (UDVA/CDVA), manifest refraction and binocular optotype-moving DVA of 40 and 80 degrees per second (dps) were evaluated pre-operatively and post-operatively up to 3 months.

**Results:**

The study included 264 eyes of 132 subjects, with an average age of 27.0 ± 6.7 years, and females accounted for 59% of the participants. Significant improvement was observed at the 3-month visit for 40 dps (SMILE, *P* = 0.001; LASEK, *P* = 0.006; FS-LASIK, *P* = 0.010) and 80 dps (SMILE, *P* = 0.011; LASEK, *P* = 0.025; FS-LASIK, *P* = 0.012) DVA. Adjusting for pre-operative DVA, there was no significant difference in DVA among groups at 3 months post-operatively (*P* > 0.05 for multiple comparisons). Overall, multiple linear models demonstrated that post-operative DVA at 3 months was correlated with pre-operative DVA (40 dps, β = 0.349, *P* = 0.001; 80 dps, β = 0.447, *P* < 0.001), pre-operative spherical equivalent (40 dps, β = 0.311, *P* = 0.003; 80 dps, β = 0.261, *P* = 0.009) and post-operative UDVA (40 dps, β = −0.224, *P* = 0.024; 80 dps, β = −0.188, *P* = 0.05).

**Conclusion:**

Dynamic visual acuity at 3 months post-operatively of the three corneal refractive surgeries was better than that before the surgery in adult myopic patients, and there was no significant difference among different surgical techniques. Post-operative DVA at 3 months was found correlated with pre-operative DVA, pre-operative SE, and post-operative UDVA. With further improvement, DVA could be a promising functional visual indicator for myopic patients undergoing refractive surgeries.

## Introduction

Laser corneal refractive surgery has become an effective alternative to refractive error correction. These types of surgery correct the refractive error by quantitatively removing corneal tissue and reshaping the cornea (Wen et al., 2017). Currently, it mainly includes three categories: corneal surface ablation techniques (such as laser-assisted sub-epithelial keratomileusis, LASEK), corneal stroma ablation surgery (such as femtosecond laser-assisted *in situ* keratomileusis, FS-LASIK), and refractive corneal lenticule extraction procedures (such as small incision lenticule extraction, SMILE) (Wen et al., 2017). Each of these types of surgery has its advantages and disadvantages. Previous studies have demonstrated that corneal surface ablation surgery exhibit better performance in visual quality–related outcomes, including higher-order aberrations (HOAs) and contrast sensitivity (CS) (Kirwan and O’Keefe, 2009; Wen et al., 2017), while corneal stromal ablation surgery, especially FS-LASIK, demonstrates relative advantages in efficacy and predictability (Wen et al., 2017). With the advent of femtosecond lasers, SMILE surgery has gradually emerged, which may have potentially better biomechanical outcomes without a corneal flap compared with LASIK (Reinstein et al., 2014; Chansue et al., 2015).

Current visual quality assessment for laser refractive surgery mainly focuses on static vision, including visual acuity, optical aberration and contrast sensitivity (Reinstein et al., 1995; Stulting et al., 2011; Yıldırım et al., 2016). Dynamic visual acuity (DVA) refers to the ability to recognize objects with relative motion (Geer and Robertson, 1993; Long and Zavod, 2002; Hirano et al., 2017). DVA is a comprehensive reflection of visual function and perceptual judgment ability. Measuring visual acuity in dynamic situations, as well as the detail resolution of moving objects, can better reflect the visual function in real-life scenes (Long and Zavod, 2002). Therefore, DVA is now gradually recognized as an important evaluation index for functional vision (Ao et al., 2014; Wu et al., 2022), with important applications in sports performance (Uchida et al., 2012), driving safety evaluation (Wilkins et al., 2013; Lacherez et al., 2014), and ocular diseases (Wu et al., 2021). The previous study demonstrated that myopia significantly affects DVA when fully corrected with spectacles, and worse DVA was associated with more significant myopia (Wang et al., 2022b). Further research showed that the uncorrected DVA post-SMILE surgery is significantly better than the pre-operative corrected DVA (Wang et al., 2022a). However, the impact of different refractive surgeries on DVA remained to be explored in a controlled study.

The present study aims to compare the influence of different corneal refractive surgeries on DVA and investigate the associated factors. Measuring the DVA before and after three corneal refractive surgeries might help us to understand the impact of the different surgeries on functional vision so that DVA can be used as a supplement to the existing indicators to guide the selection of refractive surgery based on patients’ requirements in the future.

## Materials and methods

### Subjects

The present research was a prospective case series performed following the tenets of the Declaration of Helsinki. The protocol of the study was approved by the ethics committee of Peking University Third Hospital (IRB00006761-M2020431) and informed consent was obtained from all subjects. The trial was registered at ClinicalTrials.gov (ChiCTR2000037814).

Consecutive patients undergoing bilateral SMILE, FS-LASIK or LASEK surgery were prospectively enrolled when they met the following inclusion criteria: (1) age 18 to 40 years, (2) correction of myopia or myopic astigmatism for Plano target; (3) pre-operative myopia less than 10 D, astigmatism less than 3 D and anisometropia less than 3D; and (4) pre-operative and 3-month post-operative corrected distance visual acuity (CDVA) could be fully corrected to 0 (LogMAR). Exclusion criteria were as follows: (1) history of severe ocular diseases, including keratoconus, glaucoma, retinal diseases, and severe ocular surface diseases; (2) complications including severe haze and irregular corneal topography; (3) vestibular dysfunction and extraocular muscle abnormalities that affect the free movement of eyes; and (4) cognitive disorders or other systemic diseases causing poor cooperation.

### Pre-operative and post-operative evaluation

All recruited patients underwent a detailed pre-operative evaluation, including uncorrected distance visual acuity (UDVA), CDVA with LogMAR visual chart, cycloplegic and non-cycloplegic automatic (TOPCON KR8100, Japan) and manifest refraction, slip-lamp biomicroscopy (IM 900, Köniz), non-contact tonometer (NCT NIDEK Co., Ltd), dilated funduscopy, IOL master 700 (Carl Zeiss Meditec AG), corneal topography (Pentacam, Oculus, Germany). All patients were scheduled to be examined at 1 week, 1 month, and 3 months post-operatively. Non-contact intraocular pressure, slip-lamp biomicroscopy, UDVA, and non-cycloplegic automatic refraction were evaluated at each follow-up. Non-cycloplegic manifest refraction and CDVA were measured at 1 and 3 months post-operatively.

### Dynamic visual acuity testing procedure

The DVA test procedure was consistent with previous studies (Ao et al., 2013, 2014; Liu et al., 2020; Wu et al., 2022). The optotypes were presented on a 24-inch In-Plane Switching screen with a resolution of 1920 × 1080 pixels (refresh rate of 60 Hz, brightness of 30 lux). The size and configuration of the moving optotype were designed according to the standard logarithmic visual chart, and the velocity was quantified with the viewing angle (degree) changes per second (dps). Optotype generation and presentation were controlled using Matlab2017b (MathWorks, United States).

Binocular DVA (abbreviated as DVA) of 40 and 80 dps was evaluated pre-operatively and post-operatively at each follow-up. To avoid the impact of refractive errors, pre-operative DVA was examined with CDVA spectacles. Post-operative DVA was tested with the naked eye. Subjects were required to seat 2.5 m away from the screen to provide approximately 10° visual angle. The optotype moved horizontally from left to right in the middle of the screen with a random opening direction, and subjects were required to identify the direction. The DVA test logic was similar to the static visual acuity test. The test started with the optotypes three to four sizes larger than the static visual acuity. There were eight optotypes presented once per 2 s for a certain size. One size smaller optotype would be displayed only if no less than five of eight optotypes were identified correctly. The record consisted of two parts, the minimize size (A, LogMAR) that subjects could identify five or more optotypes and the number (B) of optotypes with one size smaller that could be recognized. The result was calculated using the following equation:


$$D\,V\,A=-L\,o\,g_{10}\,A-\frac{0.1}{8}*B$$

### Surgical procedures

The surgery was selected non-randomized according to the manifest refraction, corneal topography, and patients’ intention based on sufficient informed consent. The same surgery was carried out in both eyes. All patients received standard sterile draping. After topical anesthesia using oxybuprocaine hydrochloride, the eyelids were prepared with the povidone-iodine 5% solution. The procedures of different surgery were as follows:

Laser-assisted sub-epithelial keratomileusis: Surgery began with epithelial removal after soaking in 20% ethanol for 20 s. The stroma was wiped with a sponge, and the ablation was performed by WaveLight EX500 excimer laser (Alcon Laboratories Inc., Fort Worth, TX, USA). Next, the 0.02% mitomycin C cotton pad was applied to the corneal stroma, and then the cornea was rinsed with normal saline. Finally, a bandage soft contact lens (Acuvue, Johnson Vision Care. Inc., FL, United States) was placed on the cornea.

Femtosecond laser-assisted *in situ* keratomileusis: Flaps were created by WaveLight FS200 laser (Alcon Laboratories Inc., Fort Worth, TX, USA) with a thickness of 110 μm, a diameter of 8.5 to 9.0 mm and a site-cut angle of 90°. Then corneal flaps were lifted, and the stroma bed was exposed. Ablation with WaveLight EX500 excimer laser (Alcon Laboratories Inc., Fort Worth, TX, USA) began when iris registration was activated. Afterward, the flaps were repositioned, and normal saline irrigation was used to remove debris. Finally, a slightly moistened sponge was used to ensure the flaps were in a good position with no striae.

Small incision lenticule extraction: The intrastromal lenticule was created using a 500 kHz Visumax femtosecond laser (Carl Zeiss Meditec AG, Jena, Germany) with the following parameters: cap thickness of 120 μm, cap diameter of 7.5–7.8 mm, the optical zone of 6.5–6.6 mm and laser energy of 130 nanojoules (nJ). The thickness of the lenticule was dependent on the pre-operative manifest refraction. After the anterior and posterior surfaces of the lenticule were separated bluntly, the lenticule was extracted from a 2-mm incision at 10 o’clock manually.

After surgery, all patients were given 0.5% Levofloxacin drops (Santen Pharmaceutical Co., Ltd., Osaka, Japan) and 0.5% Loteprednol Etabonate Ophthalmic Suspension (Bausch & Lomb Incorporated, FL, United States) four times a day for 1 month. The dosage of preservative-free artificial tears was adjusted according to patients’ symptoms.

### Statistical analysis

Statistical analysis was performed using IBM SPSS 26.0 (IBM, Armonk, NY, United States). The graphics were generated with Microsoft Excel (2020, Microsoft Corp). The astigmatism analysis was calculated using the Alpins method (Alpins, 1993). The sphere plus half of the cylinder diopter was equal to the spherical equivalent (SEQ). Continuous variables were shown in mean ± SD (range), and categorical variables were presented with the number (percentage). One-way analysis of variance was applied to compare the baseline clinical data, including age, sphere, cylinder, SEQ, central corneal thickness and average keratometry. Pre-operative and post-operative DVA at each visit time was compared with mixed linear models in consideration of the relevance of repeated measurements. The random effect was included for the subjects, and the time point was disposed of as the repeated factor. The post-operative DVA at each time point was also compared among groups with a linear mixed model adjusting for the pre-operative DVA. Changes in LogMAR between pre-operative and 3-month post-operative DVA were calculated. Spearman’s correlation was conducted between 3-month post-operative DVA and DVA changes and their potential influential factors, including age, pre-operative DVA, pre-operative mean binocular SEQ, post-operative UDVA, CDVA and mean binocular SEQ at 3 months. Multiple linear models were applied to analyze associated factors for post-operative DVA at 3 months. Bonferroni’s correction was conducted for multiple comparisons. *P* < 0.05 was considered statistically significant.

## Results

### Baseline clinical features

A total of 264 eyes of 132 patients (mean age 27.0 ± 6.7 years; 59% female) were included in the study. The baseline clinical characteristics are summarized in Table 1. Statistical differences existed in the pre-operative sphere, cylinder and SEQ among groups (*P* < 0.001) and *post hoc* comparison demonstrated that sphere, cylinder and SEQ were the greatest in FS-LASIK eyes (*P* < 0.001). The pre-operative central corneal thickness was 556.52 ± 27.64 μm, 528.15 ± 35.69 μm, and 545.83 ± 21.52 μm (*P* < 0.001) in SMILE, LASEK, and FS-LASIK group, respectively.

**TABLE 1:** Baseline clinical characteristics of recruited patients.

Surgery	SMILE	LASEK	FS-LASIK	*P*
Subjects (*n*)	46	40	46	
Eyes (*n*)	92	80	92	
Age (years)	27.24 ± 6.82 (18, 40)	26.10 ± 6.98 (18, 39)	26.61 ± 6.40 (18, 38)	0.734
Sphere (D)	−4.39 ± 1.10 (−6.25, −2.00)	−4.06 ± 1.24 (−7.00, −1.50)	−5.79 ± 1.70 (−9.00, −1.00)	<0.001*
Cylinder (D)	−0.56 ± 0.40 (−1.50, 0)	−0.68 ± 0.51 (−2.00, 0)	−1.36 ± 0.82 (−3.50, 0)	<0.001*
Spherical equivalent (D)	−4.67 ± 1.17 (−6.75, −2.25)	−4.40 ± 1.31 (−7.75, −1.88)	−6.47 ± 1.73 (−9.38, −1.75)	<0.001*
LogMAR CDVA	−0.06 ± 0.05 (−0.18, 0)	−0.05 ± 0.05 (−0.18, 0)	−0.06 ± 0.04 (−0.18, 0)	0.233
Central corneal thickness (μm)	556.52 ± 27.64 (507, 624)	528.15 ± 35.69 (475, 618)	545.83 ± 21.52 (503, 615)	<0.001*
Average keratometry (D)	43.11 ± 1.49 (38.79, 46.15)	43.52 ± 1.55 (40.04, 46.51)	43.43 ± 1.43 (40.28, 46.65)	0.161

CDVA, corrected distance visual acuity; D, diopter; FS-LASIK, femtosecond laser-assisted *in situ* keratomileusis; LASEK, laser-assisted sub-epithelial keratomileusis; SMILE, small incision lenticule extraction (SMILE). Continuous data are given as mean ± standard deviation (range).

*Statistically significant.

### Static vision and refraction

In this study, 98% of SMILE, 92% of LASEK, and 88% of FS-LASIK eyes achieved SEQ within 0.5 D, and the cylinder within 0.5 D was 98, 90, and 85%, respectively (Supplementary Figures 1–4). No difference was found in 3-month post-operative UDVA among groups (SMILE: 1.20 ± 0.12, LASEK: 1.16 ± 0.15, FS-LASIK: 1.19 ± 0.11, *P* = 0.195). Overall, 89% of SMILE, 79% of LASEK and 86% of FS-LASIK eyes achieved the same or better post-operative UDVA compared with pre-operative CDVA.

### Dynamic vision and refraction

**FIGURE 1:**
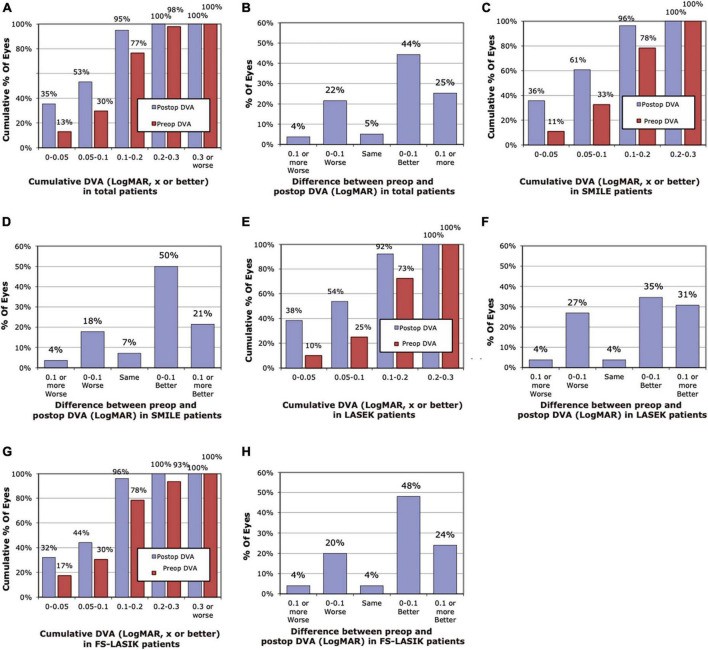
Histogram of pre-operative and post-operative dynamic visual acuity (DVA) of 40 dps at 3 months. **(A)** Cumulative pre-operative and post-operative DVA of total patients. **(B)** Changes in LogMAR between pre-operative and post-operative DVA of total patients. **(C)** Cumulative pre-operative and post-operative DVA of the SMILE group. **(D)** Changes in LogMAR between pre-operative and post-operative DVA of the SMILE group. **(E)** Cumulative pre-operative and post-operative DVA of the LASEK group. **(F)** Changes in LogMAR between pre-operative and post-operative DVA of the LASEK group. **(G)** Cumulative pre-operative and post-operative DVA of the LASIK group. **(H)** Changes in LogMAR between pre-operative and post-operative DVA of the LASIK group.

**FIGURE 2:**
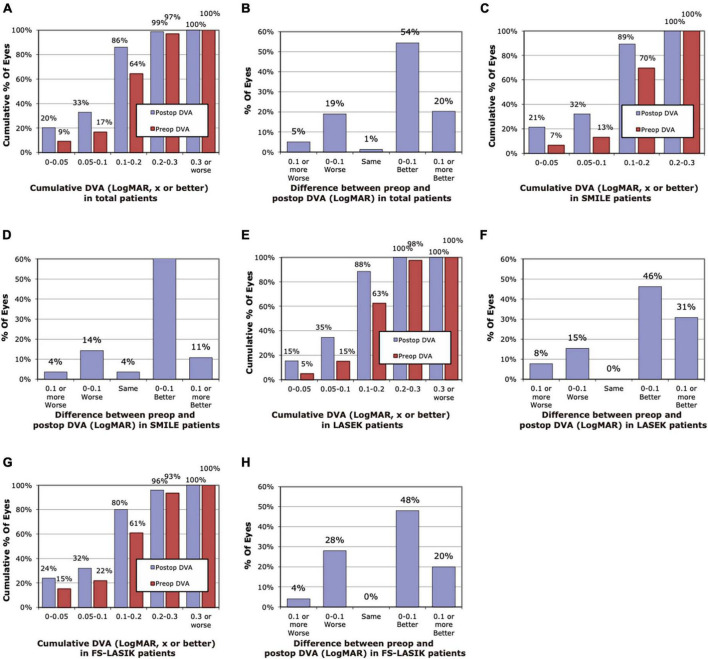
Histogram of pre-operative and post-operative dynamic visual acuity (DVA) of 80 dps at 3 months. **(A)** Cumulative pre-operative and post-operative DVA of the total. **(B)** Changes in LogMAR between pre-operative and post-operative DVA of the total. **(C)** Cumulative pre-operative and post-operative DVA of the SMILE group. **(D)** Changes in LogMAR between pre-operative and post-operative DVA of the SMILE group. **(E)** Cumulative pre-operative and post-operative DVA of the LASEK group. **(F)** Changes in LogMAR between pre-operative and post-operative DVA of the LASEK group. **(G)** Cumulative pre-operative and post-operative DVA of the LASIK group. **(H)** Changes in LogMAR between pre-operative and post-operative DVA of the LASIK group.

The histogram of pre-operative and 3-month post-operative DVA for 40 and 80 dps is demonstrated in Figures 1, 2. Compared with pre-operative DVA, more individuals achieved 0.2 LogMAR DVA at 3 months post-operatively. The percentage of individuals with better than 0.2 LogMAR pre-operative and 3-month post-operative DVA was 78 vs. 96%, 73 vs. 92%, 78 vs. 96% for 40 dps test in SMILE, LASEK, FS-LASIK groups, respectively, and that was 70 vs. 89%, 63 vs. 88%, 61 vs. 80% for 80 dps test. A total of 71, 66, and 72% of patients had better 40 dps DVA following SMILE, LASEK, or FS-LASIK at 3 months than before the surgery. As for 80 dps DVA, the percentage was 78, 77, and 68%, respectively (Figures 1, 2).

The pre-operative and post-operative DVA at each visit is shown in Table 2, and the result of multiple comparisons is demonstrated in Supplementary Table 1. No significant difference was found in pre-operative 40 and 80 dps DVA among SMILE, LASEK, and FS-LASIK groups (40 dps: 0.148 ± 0.077, 0.161 ± 0.070, 0.156 ± 0.088, *P* = 0.748; 80 dps: 0.178 ± 0.065, 0.185 ± 0.073, 0.188 ± 0.094, *P* = 0.834). At 1 week post-operatively, the patients receiving LASEK showed worse DVA (40 dps, *P* = 0.022, 80 dps, *P* < 0.001) than the pre-operative DVA measurement. Post-operatively at 1 month, a significantly improved 40 dps DVA was obtained in patients undergoing SMILE (*P* = 0.033) compared with the pre-operative DVA. Significant improvement was observed at 3 months post-operatively for 40 dps (SMILE 0.09 ± 0.068, *P* = 0.001; LASEK 0.10 ± 0.076, *P* = 0.006, FS-LASIK 0.10 ± 0.072, *P* = 0.010) and 80 dps (SMILE 0.13 ± 0.072, *P* = 0.011; LASEK 0.14 ± 0.072, *P* = 0.025, FS-LASIK 0.14 ± 0.088, *P* = 0.012) DVA compared with the pre-operative measurements.

**TABLE 2:** Mean dynamic visual acuity (DVA) and comparison among surgical methods.

	Pre-operative	1 week	1 month	3 months	*P* ^#^
**SMILE**					
Subjects (*n*)	46	46	42	28	
DVA (40 dps)	0.148 ± 0.077	0.151 ± 0.090	0.121 ± 0.066	0.093 ± 0.068	0.001*
DVA (80 dps)	0.178 ± 0.065	0.187 ± 0.090	0.170 ± 0.080	0.133 ± 0.072	0.017*
**LASEK**					
Subjects (*n*)	40	32	36	26	
DVA (40 dps)	0.161 ± 0.070	0.203 ± 0.081	0.174 ± 0.107	0.106 ± 0.076	<0.001*
DVA (80 dps)	0.185 ± 0.073	0.241 ± 0.069	0.215 ± 0.092	0.140 ± 0.072	<0.001*
**FS-LASIK**					
Subjects (*n*)	46	43	33	25	
DVA (40 dps)	0.156 ± 0.088	0.133 ± 0.085	0.145 ± 0.094	0.107 ± 0.072	0.059
DVA (80 dps)	0.188 ± 0.094	0.186 ± 0.066	0.177 ± 0.088	0.144 ± 0.088	0.056
**Total**					
Subjects (*n*)	132	121	111	79	
DVA (40 dps)	0.155 ± 0.078	0.158 ± 0.090	0.145 ± 0.091	0.102 ± 0.071	<0.001*
DVA (80 dps)	0.183 ± 0.078	0.201 ± 0.080	0.187 ± 0.088	0.138 ± 0.076	<0.001*
**Comparison among surgical methods (*P*-value^#^)**					
DVA (40 dps)	0.748	0.003*	0.033*	0.870	
DVA (80 dps)	0.834	0.004*	0.051	0.859	

dps, degree per second; DVA, dynamic visual acuity; FS-LASIK, femtosecond laser-assisted *in situ* keratomileusis; LASEK, laser-assisted sub-epithelial keratomileusis; SMILE, small incision lenticule extraction (SMILE). Data was shown as Mean ± SD.

^#^Calculated using linear mixed model, and pre-operative DVA was adjusted as a covariant when analyzes the difference in post-operative DVA among surgical methods.

*Statistically significant, *P* < 0.05.

Adjusting for the pre-operative DVA, the comparison between different surgical groups illustrated that LASEK patients presented significantly worse DVA than FS-LASIK and SMILE groups at 1-week visit (40 dps: *P* = 0.003; 80 dps: *P* = 0.004). At 1 month post-operatively, the adjusted DVA in patients undergoing LASEK was significantly worse than in patients with SMILE for 40 dps (*P* = 0.027) measurements. There was no significant difference for the adjusted post-operative DVA at 3 months among three surgical procedures (40 dps, *P* = 0.870; 80 dps, *P* = 0.859).

### Influential factors of DVA at 3 months post-operatively

Spearman’s correlation between post-operative DVA at 3 months and potentially influential factors were illustrated in Table 3. Post-operative DVA was correlated with pre-operative DVA in SMILE (40 dps: *R* = 0.488, *P* = 0.008; 80 dps: *R* = 0.728, *P* < 0.001) and FS-LASIK group (40 dps: *R* = 0.410, *P* = 0.042; 80 dps: *R* = 0.509, *P* = 0.009). A significant negative correlation was found between pre-operative SEQ and 80 dps post-operative DVA of SMILE patients (*R* = −0.374, *P* < 0.001). Post-operative UDVA was positively correlated with 40 dps DVA in the FS-LASIK group (*R* = 0.478, *P* = 0.016) and 80 dps DVA in the LASEK group (*R* = 0.501, *P* = 0.009).

**TABLE 3:** Spearman’s correlation between DVA at 3 months post-operatively and potential influential factors.

Parameters		DVA at 40 dps				DVA at 80 dps			
		**SMILE**	**LASEK**	**FS-LASIK**	**Total**	**SMILE**	**LASEK**	**FS-LASIK**	**Total**
Age (years)	R	0.029	−0.051	−0.088	−0.027	0.242	0.124	0.073	0.133
	P	0.882	0.804	0.677	0.815	0.215	0.546	0.727	0.243
Pre-operative DVA	R	0.488	0.158	0.410	0.402	0.728	0.295	0.509	0.529
	P	0.008*	0.440	0.042*	<0.001*	<0.001*	0.144	0.009*	<0.001*
Pre-operative SEQ^#^	R	−0.200	−0.201	−0.383	−0.242	−0.374	−0.137	−0.294	−0.216
	P	0.309	0.326	0.059	0.032*	<0.001*	0.504	0.154	0.056
Post-operative UDVA	R	0.270	0.320	0.478	0.353	0.364	0.501	0.297	0.403
	P	0.165	0.111	0.016*	0.001*	0.057	0.009*	0.149	<0.001*
Post-operative CDVA	R	−0.269	0.208	−0.091	−0.059	−0.185	0.409	−0.287	−0.040
	P	0.174	0.354	0.672	0.620	0.355	0.058	0.175	0.736
Post-operative SEQ^#^	R	0.065	0.144	0.299	0.160	−0.046	0.211	0.192	0.147
	P	0.747	0.524	0.156	0.177	0.821	0.346	0.369	0.215

dps, degree per second; DVA, dynamic visual acuity; FS-LASIK, femtosecond laser-assisted *in situ* keratomileusis; LASEK, laser-assisted sub-epithelial keratomileusis; SEQ, spherical equivalent; SMILE, small incision lenticule extraction (SMILE); UDVA, uncorrected distance visual acuity.

^#^Mean binocular SEQ (subjective refraction, diopter).

*Statistically significant.

Table 4 summarizes multiple linear regression results for DVA at 3 months. The regression analysis illustrated that 3-month post-operative DVA was significantly correlated with pre-operative DVA in SMILE (*P* = 0.001 for 40 dps; *P* < 0.001 for 80 dps) and FS-LASIK (*P* = 0.004 for 40 dps; *P* = 0.001 for 80 dps) groups. Post-operative DVA was correlated with UDVA at 3 months for patients undergoing LASEK (*P* = 0.005 for 40 dps; *P* = 0.001 for 80 dps) and FS-LASIK (*P* = 0.005 for 40 dps), but not for 80 dps DVA in FS-LASIK group (*P* = 0.373). Besides, a significant correlation between pre-operative SEQ and post-operative DVA at 80 dps in SMILE (*P* = 0.048) and LASEK (*P* = 0.040) groups was observed.

**TABLE 4:** Multiple linear regression for DVA at 3 months.

	Dependent variable	Independent variable	Unstandardized coefficient	Standardized coefficient	*P*	*R* ^2^	Adjusted *R*^2^
SMILE	DVA at 40 dps	Pre-operative DVA at 40 dps	0.487	0.613	0.001	0.376	0.351
	DVA at 80 dps	Pre-operative DVA at 80 dps	0.725	0.662	<0.001	0.581	0.546
		Pre-operative mean binocular SEQ^#^	−0.017	−0.279	0.048		
LASEK	DVA at 40 dps	Post-operative UDVA at 3 months	0.861	0.574	0.005	0.329	0.296
	DVA at 80 dps	Post-operative UDVA at 3 months	0.836	0.618	0.001	0.564	0.518
		Pre-operative mean binocular SEQ^#^	−0.031	−0.339	0.040		
FS-LASIK	DVA at 40 dps	Pre-operative DVA at 40 dps	0.401	0.508	0.004	0.488	0.439
		Post-operative UDVA at 3 months	1.074	0.450	0.009		
	DVA at 80 dps	Pre-operative DVA at 80 dps	0.580	0.632	0.001	0.400	0.373
Total	DVA at 40 dps	Pre-operative DVA at 40 dps	0.296	0.349	0.001	0.364	0.336
		Post-operative UDVA at 3 months	0.536	0.311	0.003		
		Pre-operative mean binocular SEQ^#^	−0.011	−0.224	0.024		
	DVA at 80 dps	Pre-operative DVA at 80 dps	0.434	0.447	<0.001	0.412	0.386
		Post-operative UDVA at 3 months	0.485	0.261	0.009		
		Pre-operative mean binocular SEQ^#^	−0.010	−0.188	0.05		

dps, degree per second; DVA, dynamic visual acuity; FS-LASIK, femtosecond laser-assisted *in situ* keratomileusis; LASEK, laser-assisted sub-epithelial keratomileusis; SEQ, spherical equivalent; SMILE, small incision lenticule extraction (SMILE); UDVA, uncorrected distance visual acuity.

^#^Mean binocular SEQ (subjective refraction, diopter).

Changes between pre- and post-operative DVA at 3 months were calculated, and its associated factors were analyzed using Spearman’s correlation, and the results are shown in Table 5. Pre-operative DVA was significantly positively correlated with DVA changes for both 40 dps (SMILE: *R* = −0.644, *P* < 0.001; LASEK: *R* = −0.457, *P* = 0.019; FS-LASIK: *R* = −0.642, *P* = 0.001) and 80 dps (LASEK: *R* = −0.531, *P* = 0.005; FS-LASIK: *R* = −0.521, *P* = 0.008), except for 80 dps DVA changes in SMILE group (*P* = 0.386). Meanwhile, age was negatively correlated with DVA changes in the SMILE group for 40 dps (*R* = −0.411, *P* = 0.030).

**TABLE 5:** Spearman’s correlation between DVA changes at 3 months and potential influential factors.

Parameters		DVA changes^a^ at 40 dps				DVA changes^a^ at 80 dps			
		**SMILE**	**LASEK**	**FS-LASIK**	**Total**	**SMILE**	**LASEK**	**FS-LASIK**	**Total**
Age (years)	R	−0.411	0.244	−0.258	−0.108	−0.289	0.083	0.131	0.025
	P	0.030*	0.229	0.214	0.343	0.136	0.688	0.533	0.824
Pre-operative DVA	R	−0.644	−0.457	−0.642	−0.597	−0.170	−0.531	−0.521	−0.415
	P	<0.001*	0.019*	0.001*	<0.001*	0.386	0.005*	0.008*	<0.001*
Pre-operative SEQ^#^	R	0.145	−0.155	−0.138	−0.088	−0.327	0.009	0.075	−0.041
	P	0.461	0.451	0.509	0.441	0.090	0.966	0.721	0.721
Post-operative UDVA	R	−0.203	0.124	0.234	0.030	−0.049	−0.010	0.129	0.044
	P	0.301	0.547	0.261	0.791	0.804	0.963	0.538	0.699

dps, degree per second; DVA, dynamic visual acuity; FS-LASIK, femtosecond laser-assisted *in situ* keratomileusis; LASEK, laser-assisted sub-epithelial keratomileusis; SEQ, spherical equivalent; SMILE, small incision lenticule extraction (SMILE); UDVA, uncorrected distance visual acuity.

^a^The DVA changes were calculated as post-operative minus pre-operative measurements, where a negative change indicated the improvement in DVA.

^#^Mean binocular SEQ (subjective refraction, diopter).

*Statistically significant.

## Discussion

Existing visual function assessment for corneal refractive surgery mainly focuses on static vision. DVA is a promising functional visual evaluation indicator. To our knowledge, this study is the first to compare DVA after SMILE, FS-LASIK, and LASEK surgeries, which can be used as a supplement to the existing indicators to guide refractive surgery visual function evaluation.

In the present study, the DVA of the LASEK group was worse than that of the FS-LASIK and SMILE groups 1 week post-operatively, and worse than that pre-operatively. During the early post-operative period, the patient receiving LASEK suffers from a corneal epithelial healing process (Ghirlando et al., 1995), and the irregular corneal surface might increase the optical scatter that induces decreased DVA. The DVA performance of the SMILE group was better at 1 month post-operatively compared with the measurement before the surgery, but a significant improvement was not observed in the other two surgeries. The result indicates that the functional vision of the patient may recover faster after SMILE than other surgeries. The previous study has demonstrated that SMILE may cause less corneal biomechanical changes at the beginning of corneal wound healing in the early post-operative stage, which may be due to stiffer anterior stroma and the corneal integrity preservation (Yu et al., 2019a), and the refractive power was more uniform from central to peripheral cornea (Yu et al., 2019b). There was no significant difference in DVA among the three surgical procedures at 3 months after surgery, indicating that after the corneal remodeling, the three surgical methods had comparable effects on DVA.

This study discovered that DVA at 3 months post-operatively was significantly better than pre-operative DVA. The result is similar to our previous research enrolling myopic patients undergoing SMILE (Wang et al., 2022a). The outcome may indicate that the correction of DVA with corneal refractive surgeries was significantly better than that of spectacles. When observing a moving object, the eye needs to keep the object relatively stable on the retina through smooth tracking and saccade, and keep the fovea’s fixation on the image as much as possible (Spering and Montagnini, 2011). Previous studies have demonstrated that the magnocellular (M) retinal ganglion cells responsible for motion perception are mainly distributed in the peripheral retina (Dacey, 1994; Skottun, 2016), and the perception of motion by the peripheral retina may affect eye tracking (Wang et al., 2021). Previous studies have shown that the resolution of the macular area to the optotype decreases with the increase of the speed of the optotype (Geer and Robertson, 1993; Lewis et al., 2011), which may be due to the macular image shift, resulting in a more obvious drag effect (Geer and Robertson, 1993). Refractive surgery and spectacles differently affect the projection of objects on the central and peripheral retina. Spectacles have obvious peripheral defocus and prism effects that cause unclear images projected to the peripheral retina (Bakaraju et al., 2008; Lin et al., 2010; Backhouse et al., 2012; Lewerenz et al., 2018; Wang et al., 2022b), and more difficulties in predicting the trajectory of the object. Thus, DVA following refractive surgeries was significantly better than the use of spectacles pre-operatively.

Our study found that post-operative DVA at 3 months was correlated with pre-operative DVA, pre-operative SE, and post-operative UDVA, which was most consistent with previous studies (Wang et al., 2022a,b). Post-operative UDVA reflects the imaging ability of the macula, which is suggested to be related to DVA. We found that worse post-operative DVA was associated with smaller pre-operative SE measurements. Combining the previous evidence that severe myopia is associated with worse DVA (Wang et al., 2022b) for myopic adults corrected with spectacles, the current result manifested that individuals with more severe myopia pre-operatively still have worse post-operative DVA though corrected with refractive surgery. Previous studies have shown that patients with severe myopia tend to have thinner retinal ganglion cell-inner plexiform layer (Mwanza et al., 2011; Seo et al., 2017; Lee et al., 2020). As mentioned above, the M ganglion cells play a key role in motion perception (Skottun, 2016). Thus, the decline in the density of M ganglion cells might lead to the decline of DVA in individuals with severe myopia. Further research is required to explore the relationship between retinal neuronal anatomy and DVA. However, we found that pre-operative DVA was positively associated with DVA changes, which indicates that patients with more severe myopia obtain more gains in DVA. The above-mentioned negative optical effect of spectacles is theoretically more obvious for higher myopia patients. Thus, correction with refractive surgery could be more beneficial in patients with higher myopia in DVA improvement.

Myopia is the most prevalent ocular disease to cause decreased uncorrected visual acuity. It could be corrected with several methods, among which spectacles are mostly used. In the present research, we found that patients undergoing laser refractive surgery achieved improved DVA compared with that before the surgery corrected with spectacles. Therefore, for individuals with higher dynamic vision demands, such as those involved in athletic competition, driving and flight task, refractive surgery might be a better corrective option than spectacles. It is noted to mention that comprehensive pre-operative evaluation is required to screen eligible candidates for refractive surgery. Previous research has shown that motion perception may be more useful for driving safety than fine vision (Moharrer et al., 2020). With further research, DVA can not only be applied to evaluate the functional vision but can also guide the selection of myopia correction methods to supplement the existing indicators.

Certain limitations exist in the present study. Firstly, the follow-up was only extended to 3 months post-operatively. The DVA might change in a long-term post-operatively. Thus, measurement at 6 months and longer after refractive surgery is required in further research. In addition, the loss to follow-up rate at 3 months post-operatively is high in the present research, which might be due to the COVID-19 global pandemic. DVA is suggested to better reflect functional vision in daily life. Thus, subjective quality of life evaluation and its association with DVA is required for post-operative refractive surgery patients in the future study. In addition, this study only focused on distant DVA, and recent research suggested that dynamic defocus curves may also be promising indicators of functional vision assessing the DVA of difference distance (Wu et al., 2022). Future research can incorporate these novel indicators to evaluate the full-distance dynamic vision better.

In summary, from the 3-month observation, the DVA following three laser refractive surgeries was better than that before the surgery in adult myopic patients. There was no significant difference among different surgical techniques for post-operative DVA at 3 months. Post-operative DVA at 3 months was found correlated with pre-operative DVA, pre-operative SE, and post-operative UDVA. The present research provided the basis for applying the DVA test as an indicator for functional vision in refractive surgery and useful information for selecting the myopia correction method in a DVA-based way.

## Data availability statement

The original data presented in this study are included in the article/Supplementary material, further inquiries can be directed to the corresponding authors.

## Ethics statement

The studies involving human participants were reviewed and approved by the Peking University Third Hospital Medical Science Research Ethics Committee. The patients/participants provided their written informed consent to participate in this study.

## Author contributions

YW contributed to the design and data collection. YG involved the data collection. YG and YL analyzed and interpreted the data and major contributors in writing the manuscript. YC and XL revised the manuscript. All authors read, commented, and approved the final manuscript.
