# Supplementary material for: The impact of different corneal refractive surgeries on binocular dynamic visual acuity
Source: Front Neurosci. 2023 Mar 3;17:1142339. doi: 10.3389/fnins.2023.1142339 (PMC10022881; doi:10.3389/fnins.2023.1142339)
Supplement: Supplementary file 1 [file Data_Sheet_1.DOCX]

**Supplementary Table 1.** Post hoc comparison between preoperative and postoperative DVA at each time point with linear mixed model

|  | Velocity | Time | Preoperative | 1 w | 1m |
| --- | --- | --- | --- | --- | --- |
| **SMILE** | 40dps | 1w | 0.831 |  |  |
|  |  | 1m | 0.033* | 0.02* |  |
|  |  | 3m | 0.001* | <0.001* | 0.128 |
|  | 80dps | 1w | 0.509 |  |  |
|  |  | 1m | 0.638 | 0.267 |  |
|  |  | 3m | 0.011* | 0.002* | 0.034* |
| **LASEK** | 40dps | 1w | 0.022* |  |  |
|  |  | 1m | 0.373 | 0.156 |  |
|  |  | 3m | 0.006* | <0.001* | 0.001* |
|  | 80dps | 1w | <0.001* |  |  |
|  |  | 1m | 0.025* | 0.144 |  |
|  |  | 3m | 0.009* | <0.001* | <0.001* |
| **FS-LASIK** | 40dps | 1w | 0.123 |  |  |
|  |  | 1m | 0.599 | 0.380 |  |
|  |  | 3m | 0.010* | 0.195 | 0.048* |
|  | 80dps | 1w | 0.961 |  |  |
|  |  | 1m | 0.700 | 0.671 |  |
|  |  | 3m | 0.012* | 0.012* | 0.042* |
| **Total** | 40dps | 1w | 0.627 |  |  |
|  |  | 1m | 0.349 | 0.169 |  |
|  |  | 3m | <0.001* | <0.001* | <0.001* |
|  | 80dps | 1w | 0.026* |  |  |
|  |  | 1m | 0.518 | 0.138 |  |
|  |  | 3m | <0.001* | <0.001* | <0.001* |

FS-LASIK = femtosecond laser-assisted in situ keratomileusis; LASEK = Laser-assisted sub-epithelial keratomileusis; SMILE = small incision lenticule extraction (SMILE); Time = follow-up time, 1w = one week after surgery onset; 1m = one month after surgery onset; 3m = three months after surgery onset;

*Statistically significant

**
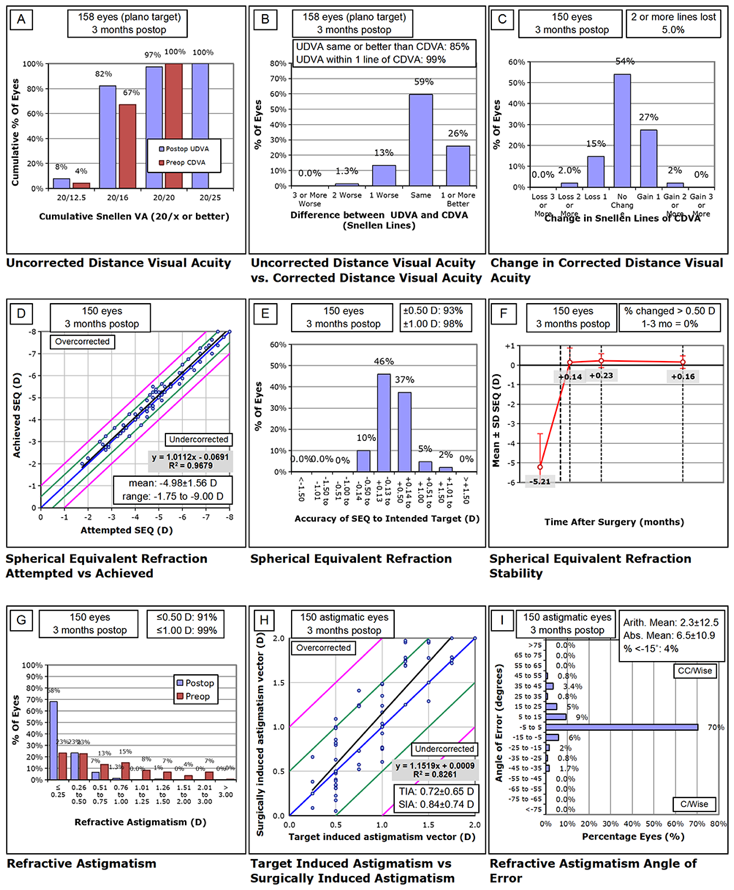
**

**Supplementary Figure 1.** Standardized graphs (9 components) of refractive outcomes of all recruited patients at 3 months:(A): cumulative Snellen visual acuity of preoperative CDVA and postoperative UDVA; (B) difference between postoperative UDVA and preoperative CDVA; (C)changes in Snellen lines of CDVA; (D) achieved spherical equivalent postoperative versus attempted SEQ; (E) SEQ refraction accuracy; (F) changes of SEQ over time; (G) preoperative and postoperative residual astigmatism; (H) target induced astigmatism versus surgically induced astigmatism; and (I) histogram of angle of error. CDVA = corrected distance visual acuity; SEQ = spherical equivelanet; UDVA = uncorrected distance visual acuity;

**
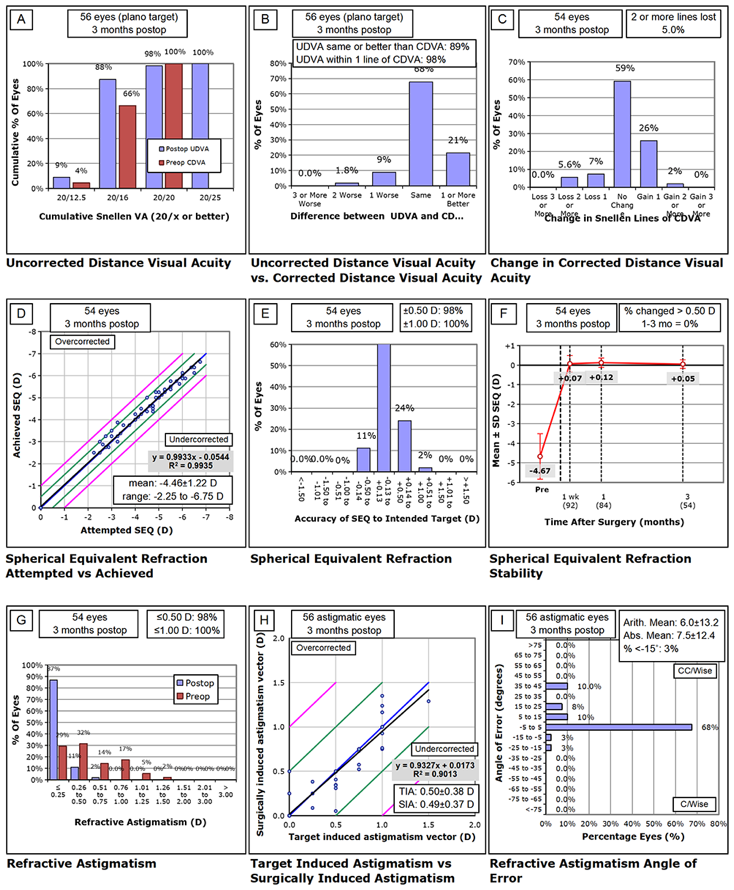
**

**Supplementary Figure 2.** Standardized graphs (9 components) of refractive outcomes of SMILE eyes at 3 months:(A): cumulative Snellen visual acuity of preoperative CDVA and postoperative UDVA; (B) difference between postoperative UDVA and preoperative CDVA; (C)changes in Snellen lines of CDVA; (D) achieved spherical equivalent postoperative versus attempted SEQ; (E) SEQ refraction accuracy; (F) changes of SEQ over time; (G) preoperative and postoperative residual astigmatism; (H) target induced astigmatism versus surgically induced astigmatism; and (I) histogram of angle of error. CDVA, corrected distance visual acuity; SEQ, spherical equivelanet; UDVA, uncorrected distance visual acuity;

**
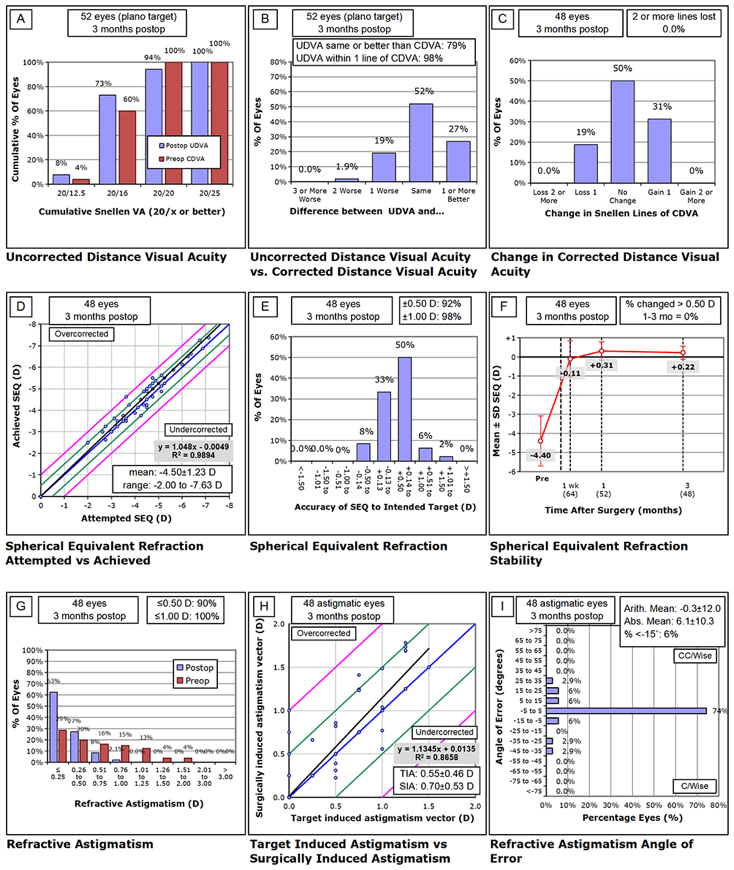
**

**Supplementary Figure 3.** Standardized graphs (9 components) of refractive outcomes of LASEK eyes at 3 months:(A): cumulative Snellen visual acuity of preoperative CDVA and postoperative UDVA; (B) difference between postoperative UDVA and preoperative CDVA; (C)changes in Snellen lines of CDVA; (D) achieved spherical equivalent postoperative versus attempted SEQ; (E) SEQ refraction accuracy; (F) changes of SEQ over time; (G) preoperative and postoperative residual astigmatism; (H) target induced astigmatism versus surgically induced astigmatism; and (I) histogram of angle of error. CDVA, corrected distance visual acuity; SEQ, spherical equivelanet; UDVA, uncorrected distance visual acuity;

**
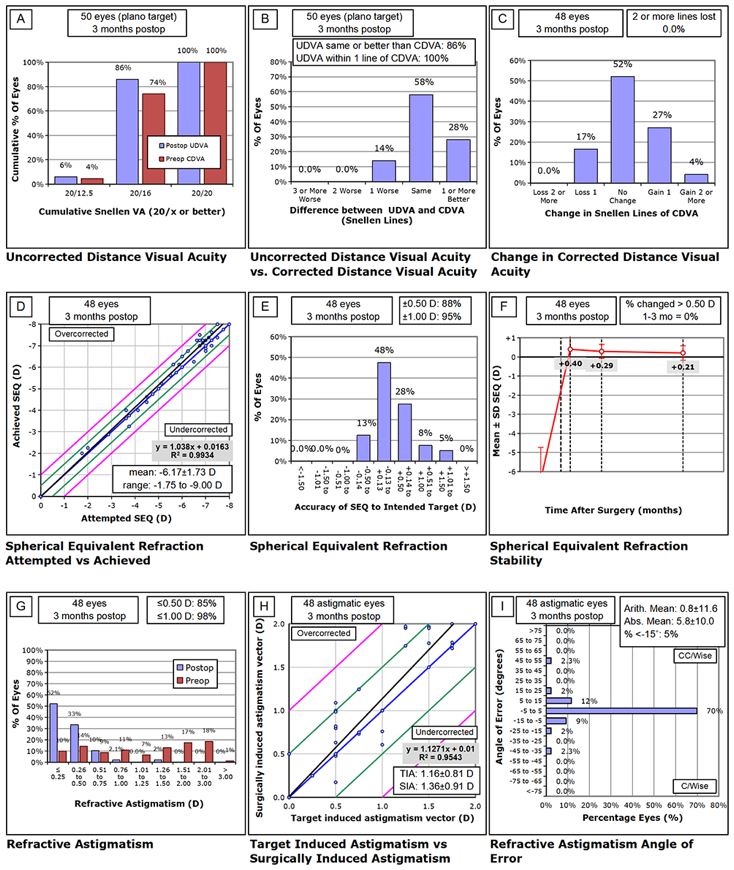
**

**Supplementary Figure 4.** Standardized graphs (9 components) of refractive outcomes of FS-LASIK eyes at 3 months:(A): cumulative Snellen visual acuity of preoperative CDVA and postoperative UDVA; (B) difference between postoperative UDVA and preoperative CDVA; (C)changes in Snellen lines of CDVA; (D) achieved spherical equivalent postoperative versus attempted SEQ; (E) SEQ refraction accuracy; (F) changes of SEQ over time; (G) preoperative and postoperative residual astigmatism; (H) target induced astigmatism versus surgically induced astigmatism; and (I) histogram of angle of error. CDVA, corrected distance visual acuity; SEQ, spherical equivelanet; UDVA, uncorrected distance visual acuity;
